# Risk behaviors of 15–21 year olds in Mexico lead to a high prevalence of sexually transmitted infections: results of a survey in disadvantaged urban areas

**DOI:** 10.1186/1471-2458-6-49

**Published:** 2006-02-27

**Authors:** Juan-Pablo Gutierrez, Stefano M Bertozzi, Carlos J Conde-Glez, Miguel-Angel Sanchez-Aleman

**Affiliations:** 1 Division of Evaluation & Health Economics, National Institute of Public Health, Cuernavaca, Mexico; 2 Division of Medical Microbiology, National Institute of Public Health, Cuernavaca, Mexico

## Abstract

**Background:**

Due to the fact that adolescents are more likely to participate in high-risk behaviors, this sector of the population is particularly vulnerable to contracting sexually transmitted infections (STIs) and resultant health problems.

**Methods:**

A survey was carried out among adolescents from poor homes in 204 small-urban areas of Mexico. Information was collected in relation to risk behaviors and socio-economic environment. A sub-group of the participants also provided blood and urine samples which were analyzed to detect sexually transmitted infections.

**Results:**

The presence of Chlamydia was detected in nearly 8% of participants who had stated that they were sexually active (18%) and approximately 12% were positive for herpes type 2-specific antibodies. For both, a greater proportion of girls resulted positive compared to boys. The presence of these biological outcomes of sexual risk behavior was associated with other risk behaviors (smoking), but not with self-reported indicators of protected sex (reported use of condom during most recent sexual activity).

**Conclusion:**

The results presented in this study show a startlingly high prevalence of HSV-2 among sexually active Mexican adolescents in poor urban areas, suggesting that this group has participated to a great extent in risky sexual practices. The relationships between socioeconomic environment and adolescent risk behavior need to be better understood if we are to design preventive interventions that modify the determinants of risk behaviors.

## Background

Adolescence is a critical period of development, as important behavior patterns are set in place during this period, and these patterns can have serious and long-term consequences for lifetime health and welfare. In particular, adoption of unhealthy risk behaviors such as smoking and drinking, and participation in others such as unprotected sex, can lead to health problems that will reduce both life expectancy and quality of life.

Globally, adolescents face many problems related to sexually transmitted diseases including HIV/AIDS, unwanted pregnancy and drug, alcohol, and tobacco use. As countries develop and poverty rates fall, either because of economic growth or explicit poverty alleviation programs, adolescents living in poor households will obtain the knowledge and resources to be able to reduce their risks; however, they will also have more opportunities and resources to engage in these risky behaviors.

The World Health Organization (WHO) estimates that 1 in 20 adolescents throughout the world will contract a curable sexually transmitted infection (STI) (without taking into consideration viral infections) [1]. This number illustrates the importance of preventing risk behaviors among adolescents, in particular those related to sex, especially if we consider that those who contract sexually transmitted infections are just a small percentage of those who are exposing themselves to infection through unprotected sex.

One of the most common viral STIs is herpes simplex type 2 (HSV-2) [2], which has also been associated with a higher probability of HIV transmission [3,4]. The literature suggests that the presence of HSV-2 most commonly goes undetected by carriers. In particular, one study of adolescents in the United States discovered that only 22% of positive HSV-2 cases reported a prior history of herpes [5]. Thus, measuring the prevalence of herpes antibodies is a far better measure of infection rates and, since it is (one of) the most prevalent STIs, at least in Mexico, it is also important as a marker of sexual risk behavior [6-9]. Furthermore, said behaviors may also lead to unwanted pregnancy and its consequences.

In Mexico, problems associated with sexual activity leading to unwanted pregnancies and sexually transmitted infections, as well as alcohol and tobacco use, are a major concern, particularly among adolescents living in poor households. In 2003, 17% of all births in Mexico were to women under the age of 20 [10]. As far as bacterial STIs are concerned, probably the most studied infections are Chlamydia and gonorrhea, for which information relating to the general population suggests that the prevalence of Chlamydia is low and that of gonorrhea is very low [11-14].

There are very few figures relating to STIs among adolescents in Mexico and, in general, they are limited to one region or federal state. Therefore, very little is known about the prevalence of this type of infection in this age group [15,16]. Of published studies, one study carried out in Morelos discovered a 5.7% prevalence of HSV-2 among sexually active adolescents and youth in school between the ages of 11 and 24, with a female to male ratio of 2 to 1, and a peak among sexually active high school students [17].

Furthermore, it has been suggested that risk behaviors occur in groups, i.e. the probability of risk behavior occurring is higher if another risk behavior has already occurred [18].

The aim of this paper is to present evidence from a national survey among adolescents living in poor small urban areas, describing both prevalence of risky behaviors and their relationship with poverty.

## Methods

As part of the evaluation of an antipoverty program in Mexico, a survey was carried out in a sample of 23,000 homes located within poor census tracts (AGEBs, as defined by Mexican Population Council) in 204 towns with between 2,500 and 75,000 inhabitants (considered to be small urban towns in Mexico). The sample was selected to represent households eligible to receive the program's benefits, with an additional sampling of non-eligible households, selected to test targeting. To identify eligible households, the program identified census tracts with the highest prevalence of poverty from the 2000 national census within towns that had access to basic health and education services. The program conducted a preliminary survey of all households within the selected census tracts, collecting information about household members, household assets and housing characteristics which was used to generate a poverty score that determined. The towns to be included in the program defined the universe for the sample. Sampling was multi-staged. Towns were selected randomly with probability proportional to size. Then, within towns, eligible and non-eligible households were randomly selected.

As a part of the survey, a specific questionnaire was administered to 15 to 21 years old and blood and urine samples were collected from the same. The questionnaire was based on others used in Mexico for national surveys, in particular the National Health Survey and the National Fertility Survey, as well as questionnaires developed by FHI/UNAIDS and by the authors to evaluate the impact of HIV prevention programs. The instrument is available as an additional file [see Additional file 1].

For the analyses presented here we took advantage of the entire sample (including non-eligible households). We used data from the general household survey on household socioeconomic status as well as data from the adolescent survey on characteristics of the individual adolescent and his/her participation in risk behaviors. The adolescent questionnaire was administered by a trained interviewer (low literacy levels in this very poor population limit the utility of self-administered questionnaires and computer-assisted self interviewing).

We used a broader definition for adolescents, i.e. individuals from 15 to 21 years old. We acknowledge that 21 is old with respect to usual definitions, but the behaviors that we are collecting information about are largely behaviors that began in the past, so participants are reporting on behaviors over prior (adolescent) years.

The design of the study was approved by the IRB at the National Institute of Public Health (INSP). Informed consent was obtained from parents or guardians for all underage participants, and directly for 18 to 21 years old participants.

### Adolescent sample

The adolescent component of the survey was designed for the 15- to 21-year-olds in the population. It consisted of a questionnaire and the collection of urine and blood samples, to measure the prevalence of sexually transmitted infections (STIs). STI data were collected because they are important health outcomes in their own right, as well as markers of risky sexual behavior.

As the main purpose of the survey was to collect household-level data, up to three visits were made to each household and only adolescents present at one of the visits were interviewed. While some data were collected for the non-present adolescents from a proxy informant, none of the data on risk behavior could be collected using these informants and therefore these adolescents were excluded from the analysis.

Information collected from adolescents included basic demographics, schooling and labor data, as well as health related behavior such as smoking, drinking, sexual activity, condom use, and participation in compensated sex (all of the risk behavior questions were dichotomous).

### Socioeconomic indicators

The household questionnaire included information on family members and socioeconomic characteristics. In order to estimate household socioeconomic level, data on monthly monetary consumption was estimated by adult equivalent using data on food and non-food consumption from the household survey.

We compared these estimates with nationally representative consumption surveys to classify households into socioeconomic quintiles.

### Biomarker collection and analysis

After consent was obtained from parents of adolescents under the age of 18 and from the adolescents themselves, blood samples were drawn by trained phlebotomists and the adolescents were asked to provide a sample of urine. All samples were sent to the National Public Health Institute facilities, where the sera and the urine were frozen at -20 degrees centigrade until they were analyzed.

The urine samples were analyzed for the presence of Chlamydia and gonorrhea using ligase chain reaction amplification (LCx, Abbott Laboratories) according to the manufacturer's protocol.

The blood samples were analyzed using an Elisa test (Focus Technologies) to detect the presence of anti-herpes simplex type 2 virus antibodies, following the procedures indicated by the manufacturer of the tests.

All samples were analyzed in the National Institute of Public Health by trained technicians.

### Statistical analysis

Biomarker data were merged with the individual data using a unique, pre-assigned identification code. All biomarker data could be matched with the socioeconomic and behavioral information.

All variables included in the analysis reported here were revised to check for inconsistent, impossible, or missing values. To the extent possible these were recoded to the mean or more appropriate value when available information permitted, so as not to have to exclude the entire observation. These data-cleaning procedures affected less than 5% of the observations.

Descriptive statistics and all analysis were estimated using Stata 8.0 (Stata Corporation). We Standard errors and confidence intervals are adjusted for design effect, considering both strata and primary sampling units (PSU). Sample weights were also employed.

HSV-2 serostatus and Chlamydia infection were included as dichotomous variables and prevalences were estimated by age group (as defined below) and sex.

To estimate the ability of socioeconomic variables to predict risk behaviors, we performed a set of multivariate logistic regressions, using each of the dichotomous indicators of risk behaviors (tobacco, alcohol, ever having had sex, condom use, prior participation in compensated sex, HSV-2, and Chlamydia) as dependent variables. All dependent variables corresponded to a specific yes/no question included in the survey or the result of a laboratory test.

In addition to the household socioeconomic status defined above, we included as explanatory variables: age, sex, educational attainment, perceived social support, labor force participation, and whether the adolescent was living with a partner. In predicting condom use at last sex and compensated sex among the sexually active, we included, in addition to the above, years since first sexual intercourse, smoking, drinking, and history of reported STI symptoms. In predicting infection with HSV-2 and Chlamydia, we additionally included condom use at last sex and participation in compensated sex.

Age was categorized into two groups (15–18, 19–21), and years since first sexual intercourse into three categories (≤ 1 year, 2–3 years, and ≥ 4 years). Labor force participation and marital status were categorized as yes/no variables. Marital status was coded as 1 if the adolescent was cohabitating with a partner, whether married or not.

For schooling, we included three different variables: whether the adolescent was attending school at the time of the survey, whether they were older than predicted by their last completed grade in school (coded as 1 if not older), and, if they were older, the difference in years between actual and predicted age. In this way, we differentiated the effect of educational achievement from the peer group effect associated with attending school.

Having social support was defined as having a friend in whom they could confide their personal problems.

## Results

A total of 13,897 adolescents were living in the selected households at the time of the survey. Of these, socioeconomic and behavioral data were collected from 13,444 (97%), with the great majority of the remainder absent at the time of the visits. Blood and/or urine samples were collected from the 4,024 adolescents who agreed to provide them. Response was partially limited by the adolescents' refusal to provide a sample, but was more a function of the fact that the biological field teams were understaffed in comparison to the socioeconomic teams and thus requested samples from a subset of the adolescents. While samples were obtained from only 29% of the adolescents, all observable variables (age, male-female distribution, percentages of adolescents who go to school and who work) are similar in the group that agreed to give just urine, just blood or both, compared with the total sample. Out of all of the complete samples collected, only those from adolescents who reported having been sexually active were analyzed, as well as small proportion of urine samples of those reported been non-sexually active. A total of 753 blood samples and 1,620 urine samples were analyzed (1,241 from sexually active). The main characteristics of the participants in the survey can be found in Table 1. Table 2 shows the specific characteristics of those who reported that they had been sexually active. To estimate the degree of under-reporting of sexual activity, we tested the urine for Chlamydia of a sub-sample of those who reported not being sexually active. While not directly comparable because of differences in sample weights, the prevalence of Chlamydia among those who report that they are not sexually active was approximately one third of that among those who report sexual activity.

The average age of all sexually active adolescents (18.7) was similar to the average age of adolescents who provided blood samples (18.8) and urine samples (18.4).

More girls than boys reported that they were sexually active, which is possibly due to the fact that the likelihood of finding girls during the survey was greater (they spend more time at home).

Table 1 shows that sexual activity is inversely correlated with being in school and is directly correlated with working. School attendance is higher among girls who did not report sexual activity between the ages of 15 and 18 years (47%) and drops to 3–4% among sexually active girls in the two age groups.

As far as the consumption of addictive substances is concerned, a direct association was also observed between the prevalence of this behavior and sexual activity: the percentage of those surveyed who reported having smoked at some time is between 50% and 100% higher among those who are sexually active than among those who did not report sexual activity. The same was also true, although to a lesser extent, for the consumption of alcohol and marihuana.

The percentage participating in any violent event or receiving social support was similar whether or not the subjects were sexually active. As far as sexual risk behaviors are concerned, participation in compensated sex was similar across age groups and sexes, with an average of 2% for both sexes and age groups. For participation in compensated sex, the survey was not able to distinguish between buyers and sellers and it is likely that the pattern of participation is different between boys and girls. As was expected, length of time since beginning sexual activity was greater for 19- to 21-year-olds compared with 15- to 18-year-olds, the average being greater for girls in the older age group, and for boys in the younger age group.

The proportion of boys using condoms during the most recent sexual act was far greater than that of girls in both age groups, and the proportion was lower in the 19- to 21-year age group than in the 15- to 19-year age group. A very low percentage of adolescents reported having experienced symptoms of an STI during the previous 12 months.

Sixty of the 1,241 urine samples from sexually active adolescents tested positive for Chlamydia and only one for gonococcus. Using sample weights and adjustment according to type of sample, the estimated prevalence of Chlamydia was 8% among sexually active adolescents. However, as presented in Table 2, this percentage was not homogenous, ranging from 4% in 19- to 21-year-old boys to 10% in 15- to 18-year-old girls.

Furthermore, 102 of 753 blood samples resulted positive for herpes simplex type 2 virus specific antibodies, which, after adjustment, represents a prevalence of 12%. In this case, significant diversity was also found, with a prevalence of 4% in 15- to 18-year-old girls, and a maximum of 19% in 19- to 21-year-old girls.

### Variables associated with risk behaviors and STIs

Tables 3, 4, and 5 show the results of a series of multivariate logistic regression models used to explore associations between participation in risk behaviors and STIs, and characteristics of the individual and his/her household. The odds ratios from the regressions were transformed into prevalence ratios to avoid overinterpretation of the results, following the formulae suggested by Zhang & Yu [19].

As presented in Table 3, consumption of tobacco and alcohol are positively associated with male sex as has been reported in other surveys in the country. With respect to socioeconomic level, while a higher prevalence of drinking is associated with living in households that fall in the top three socioeconomic quintiles, the same relationship could not be demonstrated for tobacco. The latter does not confirm the results from past national surveys that have reported a strong positive association between socioeconomic status and smoking. However, both attending school and age-corrected education level were associated with lower prevalence for smoking but not for drinking.

Participating in the labor force was positively associated with drinking but not with smoking. Some geographical differences were also observed, as presented in the table.

In regard to initiation of sexual activity, attending school, age-appropriate education level, and participating in the labor force were all associated with delayed initiation. As expected, older adolescents and adolescents living with a partner are more sexually active. The proportion that was sexually active also increased with household socioeconomic level.

For sexually active adolescents, two additional behavioral models were estimated to predict condom use in the last sexual intercourse and participation in compensated sex. Men were more likely to use condoms and adolescents living with partners less likely, as expected given Mexican cultural norms. Of the remaining independent variables only socioeconomic status was significantly predictive of condom use: the higher the socioeconomic level, the greater the probability of using a condom.

Having a close friend was strongly protective against participation in compensated sex.

Finally, two models were estimated to predict HSV-2 and Chlamydia infection. The probability of infection with both STIs was dramatically greater for girls than boys and in the case of HSV-2, increased with age. The latter difference is not surprising, because the HSV-2 test measures antibodies which reflect cumulative life-time incidence, while in the case of Chlamydia, the test measures current active infection. Participation in compensated sex was associated with increased likelihood of being positive for both STIs, as was smoking, though the association was statistically significant only for Chlamydia.

## Discussion

The panorama presented here of Mexican adolescents in poor areas is not an encouraging one. Unhealthy behaviors are broadly prevalent and there is at least a significant risk that economic development may worsen the situation. Poverty alleviation programs, if not well designed, can make it easier for adolescents to engage in unhealthy behaviors, thus highlighting the importance of both understanding how poverty and development affect risk behaviors, and of assessing the impact of poverty alleviation programs on those same behaviors. We observed a positive relationship between socio-economic level and sexual activity in these cross-sectional data. While this does not mean that socioeconomic development will lead to increased risk, it does heighten the concern.

The results presented in this study show a startlingly high prevalence of HSV-2 among sexually active Mexican adolescents in poor urban areas. Although previous small-scale studies have been carried out that suggest that HSV-2 is highly prevalent among Mexican adolescents, this study, which sampled adolescents from 28 of the 32 Mexican states, offers a far more comprehensive overview of the problem, at least among adolescents in poor homes. The prevalence of HSV-2 found in this study is greater than that observed among young university students in the State of Morelos, a group with an average age greater than those interviewed here, which would suggest that infections are occurring at an ever earlier age.

The high prevalence of viral STIs suggests that this group has participated to a great extent in risky sexual practices, which may have implications not just for sexually transmitted infections, but also for unwanted pregnancies and their consequences. Although the current prevalence of HIV among adolescents in Mexico is very low, this also suggests that there is serious potential for spread of HIV in this population.

The other important result is the lack of correlation between self-reported risk behavior and HSV-2 serostatus. This calls calls into serious question studies that rely exclusively on self-reported behavior, as well as the assumption that there is necessarily a strong correlation between self-reported individual risk behavior and that individual's infection status. There are several possibilities that may help to explain the lack of association. One obvious possibility is that adolescents may not respond truthfully regarding condom use. While always a possibility, it seems unlikely that this explanation is sufficient because it suggests that they are untruthful in both directions. More likely, it is a combination of two other factors. First, if there is a lot of variability among adolescents in condom use from one sex act to another, then reported condom use at last sex would be a poor predictor of an individual's history of condom use. To the extent that the quantity/frequency of sexual experience is highly variable, it could further mask the association. [20] Second, probability of infection with an STI may be far more determined by the prevalence of that STI in the micro-environment from which an adolescent selects his/her sexual partner(s) than by an individual's own number of partners and condom use.

The study also highlights the link between sexual behavior and other risk practices. Although a causal relationship between smoking and the presence of an STI is not suggested, reported links suggest that adolescents who participate in one risk behavior are more likely to participate in others. Similarly, the clear link between HSV-2 and participation in compensated sex suggests that this practice may make male and female adolescents in Mexico highly vulnerable to infections, although the limited size sample needs to be taken into consideration.

The present analysis is not able to disentangle the mechanisms through which poverty, schooling, and other aspects of development affect individual risk behaviors. For example, we observed a strong positive correlation between smoking and sexual risk behavior and a strong inverse association between school achievement and smoking. Schooling, whether because of the education or the environment it provides, may have a broad impact on a large class of risk behaviors, which in our model is captured by the association with smoking. It may also be indirectly affecting sexual risk behavior, but the effect is masked in the model.

Finally, one point to consider is the differences between boys and girls: although boys participate more in non-sexual risk behaviors, the probability of girls using condoms is less than for boys, which translates into a higher probability of becoming infected with an STI. It is highly probable that this is an indication of increased vulnerability on two levels: on the one hand, physiological, in terms of susceptibility to infections and on the other, social, in terms of the ability to negotiate the use of a condom.

It is important to recognize the limitations of the results presented here. First, as the sample was selected to represent beneficiaries of an antipoverty program it is not representative of all adolescents in Mexico living in poor localities. Also, the response rate for biomarkers was far lower than ideal. Nevertheless, there is not an obvious bias that would suggest overestimation of the prevalence of risk behaviors. On the contrary, we might expect that adolescents with higher levels of risk behaviors would be less likely to be encountered by the survey teams in their parent's households. They might also be less willing to cooperate with government surveyors, with both effects resulting in underestimation of the true prevalences of risk behaviors and STIs.

## Conclusion

These data in some ways cause us to ask more questions than they answer. The relationships between absolute poverty, relative poverty, education, and adolescent risk behavior need to be better understood if we are to design preventive interventions that modify the determinants of risk behaviors. As a global community we have been strikingly unsuccessful at changing infection rates in this population, in part because of simplistic assumptions regarding the link between knowledge and behavior. We need to further disaggregate the adolescent population in order to identify sub-populations at higher risk and the factors that explain the large differences between the sexes and between geographic areas. We need to understand why self-reported behavior is such a poor predictor of infection.

## Competing interests

The author(s) declare that they have no competing interests.

## Authors' contributions

JPG conceived the idea of this report, contributed to the design of the study, designed and led the analysis and interpretation for this report, and drafted the manuscript. SMB contributed to the idea of this report and the design of the study, and to the design, interpretation and drafting of this manuscript. CJC and MAS coordinated the analysis of the samples and to the analysis and interpretation for this report. All named authors read, commented on and approved the final version of the manuscript.

## Pre-publication history

The pre-publication history for this paper can be accessed here:



## Supplementary Material

Additional File 1 Survey questionnaire. The English translation of the questionnaire is presented in a PDF file. Click here for file

## Figures and Tables

**Table 1:** Characteristics of participants according to age group, sex, and sexual activity: mean (standard error).

	**15 to 18 years old**			
	**Boys**		**Girls**	
	**Not active**	**Active**	**Not active**	**Active**
Age (years)	16.33 (0.03)	17.01 (0.08)	16.25 (0.03)	16.87 (0.11)
N	4047	313	3888	598
Go to school (%)	43% (2%)	27% (5%)	47% (2%)	3% (1%)
N	3868	303	3732	567
Work (%)	55% (2%)	68% (5%)	34% (2%)	17% (2%)
N	3960	312	3830	596
Work and study (%)	12% (1%)	11% (3%)	10% (1%)	6% (3%)
N	3429	256	2783	130
Smokes (%)	12% (1%)	35% (4%)	3% (1%)	6% (2%)
N	2298	289	2785	576
Drinks alcohol (%)	19% (2%)	42% (4%)	9% (1%)	12% (2%)
N	2301	289	2789	578
Have tried marihuana (%)	2% (1%)	9% (4%)	0% (0%)	0% (0%)
N	2299	290	2787	579
Have participated in violent activities (%)	5% (1%)	7% (2%)	4% (1%)	3% (1%)
N	2292	289	2781	578
Receive support (%)	73% (2%)	72% (5%)	80% (1%)	72% (73%)
N	2295	290	2784	579
Have had sex (%)	8% (1%)		15% (1%)	
N	4360		4486	
	**19 to 21 years old**			
	**Boys**		**Girls**	
	**Not active**	**Active**	**Not active**	**Active**
Age (years)	19.94 (0.04)	20.03 (0.09)	19.93 (0.03)	19.99 (0.05)
N	1719	381	1611	887
Go to school (%)	13% (1%)	9% (2%)	16% (2%)	4% (1%)
N	1578	368	1508	834
Work (%)	80% (2%)	80% (4%)	46% (3%)	20% (2%)
N	1666	380	1577	886
Work and study (%)	4% (1%)	5% (2%)	10% (2%)	6% (3%)
N	1447	332	910	191
Smokes (%)	20% (20%)	35% (4%)	2% (1%)	3% (1%)
N	677	365	1056	860
Drinks alcohol (%)	34% (3%)	46% (5%)	11% (2%)	12% (2%)
N	681	366	1060	860
Have tried marihuana (%)	5% (2%)	3% (1%)	0% (0%)	0% (0%)
N	680	366	1059	860
Have participated in violent activities (%)	6% (2%)	8% (2%)	3% (1%)	5% (1%)
N	680	365	1062	865
Receive support (%)	75% (3%)	84% (3%)	82% (2%)	76% (2%)
N	678	366	1062	862
Have had sex (%)	18% (1%)		40% (2%)	
N	2100		2498	

**Table 2:** Characteristics of sexually active participants according to age group and sex: mean (standard error).

	**15 to 18 years old**		**19 to 21 years old**	
	**Boys**	**Girls**	**Boys**	**Girls**
Participation in paid sex (%)	2% (1%)	2% (1%)	1% (1%)	2% (1%)
N	313	598	381	887
Length of time sexually active (years)	1.88 (0.19)	1.65 (0.07)	3.02 (0.13)	3.57 (0.13)
N	306	589	380	886
Use of condom during most recent sexual activity (%)	36% (4%)	14% (2%)	34% (4%)	10% (2%)
N	313	598	381	887
Reported symptoms of an STI (%)	3% (2%)	1% (1%)	2% (1%)	4% (1%)
N	303	592	365	882
Positive for HSV 2 antibodies (%)	9% (3%)	4% (2%)	9% (4%)	19% (4%)
N	164	148	183	258
Positive for Chlamydia (%)	7% (3%)	10% (3%)	4% (2%)	9% (3%)
N	191	315	223	507

**Table 3:** Multivariate prevalence ratios^a ^(CI95%) for smoking, drinking alcohol and sexually activity^b^

**Variable**	**Categories**	**PR of smoking**	**PR of drinking**	**PR of sexually active**
**Age**	15 – 18	1.0	1.0	1.0
	19 – 21	0.931 (0.804 – 1.051)	1.152 (1.049 – 1.245)***	1.298 (1.153 – 1.439)***
**Sex**	Women	1.0	1.0	1.0
	Men	3.101 (2.489 – 3.621)***	1.781 (1.538 – 2.003)***	0761 (0.671 – 0.856)
**Socioeconomic level**	Lowest 20%	1.0	1.0	1.0
	Second lower 20%	0.981 (0.903 – 1.040)	1.006 (0.956 – 1.047)	1.236 (1.003 – 1.507)**
	Remaining 60%	1.018 (0.985 – 1.040)	1.034 (1.009 – 1.052)**	1.541 (1.117 – 2.101)***
**Adolescent attending school**	No	1.0	1.0	1.0
	Yes	0.759 (0.592 – 0.896)***	0.957 (0.836 – 1.058)	0.447 (0.325 – 0.606)***
**Education level at standard for age**	No	1.0	1.0	1.0
	Yes	0.932 (0.859 – 0.987)**	1.002 (0.932 – 1.060)	0.529 (0.391 – 0.707)***
**Educational gap of adolescents not attending school** ** and with education level not standard for age**	0	1.0	1.0	1.0
	Years of gap	0.993 (0.981 – 1.005)	0.997 (0.986 – 1.007)	1.000 (0.994 – 1.002)
**Report labor activities**	No	1.0	1.0	1.0
	Yes	1.115 (0.901 – 1.347)	1.314 (1.075 – 1.550)***	0.700 (0.619 – 0.785)***
**Attending school and report labor activities**	No	1.0	1.0	1.0
	Yes	1.028 (0.989 – 1.046)	0.977 (0.899 – 1.024)	1.266 (0.782 – 1.999)
**Living with a partner**	No	1.0	1.0	1.0
	Yes	1.006 (0.902 – 1.082)	0.963 (0.894 – 1.024)	3.241 (2.854 – 3.616)***
**Count with a close friend**	No	1.0	1.0	1.0
	Yes	1.016 (0.762 – 1.316)	0.924 (0.787 – 1.075)	0.965 (0.806 – 1.145)
**Geographical area of residence**	Center	1.0	1.0	1.0
	Golf	0.922 (0.832 – 0.994)**	0.933 (0.845 – 1.003)*	1.264 (1.016 – 1.548)**
	North	1.078 (0.873 – 1.228)	0.998 (0.893 – 1.080)	1.131 (0.919 – 1.375)
	North Pacific	0.990 (0.929 – 1.021)	1.001 (0.981 – 1.015)	1.389 (1.049 – 1.828)***
	Central Pacific	1.024 (0.862 – 1.131)	1.147 (1.049 – 1.220)***	1.204 (0.935 – 1.530)
	South Pacific	0.876 (0.723 – 0.969)***	0.914 (0.798 – 0.990)**	1.018 (0.801 – 1.279)
	Yucatan peninsula	0.980 (0.958 – 0.995)***	0.998 (0.976 – 1.013)	0.909 (0.679 – 1.213)
Nc		9168	9186	13,197

^a ^Estimated using the formulae PR = OR/[(1-P0)+(P0*OR)] as suggested by Zhang & Yu [18]. ^b ^Confidence intervals were estimated reflecting design effect. ^c ^Number of observations is less than in descriptive because of missing values. ***P-value < 1%; **P-value < 5%; * P-value < 10%

**Table 4:** Multivariate prevalence ratios^a ^(CI 95%) for condom use and participation in compensated sex^b^.

**Variable**	**Categories**	**PR of condom use**	**PR of compensated sex**
**Age**	15 – 18	1.0	1.0
	19 – 21	1.024 (0.818 – 1.228)	1.446 (0.607 – 2.149)
**Years of sexual activity**	< 1 year	1.0	1.0
	2 to 3	1.008 (0.812 – 1.184)	0.530 (0.141 – 0.932)***
	4 or more	0.018 (0.837 – 1.152)	0.700 (0.291 – 1.241)
**Sex**	Women	1.0	1.0
	Men	1.744 (1.534 – 1.912)***	0.928 (0.586 – 1.139)
**Socioeconomic level**	Lowest 20%	1.0	1.0
	Second lower 20%	1.101 (1.002 – 1.173)**	0.875 (0.487 – 1.058)
	Remaining 60%	1.062 (0.969 – 1.106)	0.929 (0.480 – 1.025)
**Adolescent attending to school**	No	1.0	1.0
	Yes	0.963 (0.741 – 1.099)	0.961 (0.672 – 1.046)
**Education level standard for age**	No	1.0	1.0
	Yes	0.927 (0.762 – 1.025)	1.007 (0.737 – 1.072)
**Educational gap of adolescents not attending school** ** and with education level not standard for age**	No gap	1.0	1.0
	Years of gap	1.011 (0.997 – 1.024)	0.994 (0.942 – 1.037)
**Report labor activities**	No	1.0	1.0
	Yes	0.861 (0.586 – 1.153)	1.035 (0.700 – 1.262)
**Attending school and report labor activities**	No	1.0	
	Yes	1.072 (1.009 – 1.093)**	
**Living with a partner**	No	1.0	1.0
	Yes	0.686 (0.511 – 0.864)***	0.808 (0.317 – 1.354)
**Count with a close friend**	No	1.0	1.0
	Yes	1.197 (0.785 – 1.729)	0.377 (0.098 – 0.987)**
**Drinks alcohol**	No	1.0	1.0
	Yes	0.923 (0.772 – 1.050)	0.875 (0.459 – 1.092)
**Smokes**	No	1.0	1.0
	Yes	0.991 (0.838 – 1.097)	0.969 (0.618 – 1.073)
**Reported STI symptoms**	No	1.0	1.0
	Yes	0.982 (0.917 – 1.005)	0.999 (0.865 – 1.015)
**Geographical area of residence**	Center	1.0	1.0
	Golf	1.082 (0.950 – 1.177)	1.033 (0.642 – 1.224)
	North	1.061 (0.908 – 1.157)	1.102 (0.512 – 1.319)
	North Pacific	0.987 (0.929 – 1.015)	0.942 (0.620 – 0.995)***
	Central Pacific	1.046 (0.961 – 1.099)	1.183 (0.897 – 1.290)
	South Pacific	1.104 (1.012 – 1.161)**	0.970 (0.685 – 1.049)
	Yucatan peninsula	1.003 (0.976 – 1.015)	1.022 (0.901 – 1.046)
N^c^		2067	2347

^a ^Estimated using the formulae PR = OR/ [(1-P0)+(P0*OR)] as suggested by Zhang & Yu [18]. ^b ^Confidence intervals were estimated reflecting design effect. ^c ^Number of observations is less than in descriptive because of missing values. ***P-value < 1%; **P-value < 5%; * P-value < 10%

**Table 5:** Multivariate prevalence ratios^a ^(CI95%) for Chlamydia and HSV-2 infection^b^

**Variable**	**Categories**	**PR of Chlamydia**	**PR of HSV-2**
**Age**	15 – 18	1.0	1.0
	19 – 21	0.892 (0.524 – 1.307)	2.023 (1.167 – 2.940)***
**Years of sexual activity**	< 1 year	1.0	1.0
	2 to 3	1.096 (0.856 – 1.259)	1.133 (0.766 – 1.397)
	4 or more	1.202 (0.675 – 1.566)	0.994 (0.594 – 1.349)
**Sex**	Women	1.0	1.0
	Men	0.533 (0.239 – 0.881)***	0.446 (0.221 – 0.765)***
**Socioeconomic level**	Lowest 20%	1.0	1.0
	Second lower 20%	0.991 (0.823 – 1.086)	0.885 (0.685 – 1.001)*
	Remaining 60%	1.090 (0.884 – 1.148)	1.009 (0.848 – 1.087)
**Adolescent attends school**	No	1.0	1.0
	Yes	1.036 (0.742 – 1.123)	0.995 (0.712 – 1.048)
**Education level appropriate for age**	No	1.0	1.0
	Yes	0.861 (0.393 – 1.029)	0.989 (0.770 – 1.012)
**Educational gap for adolescents not attending school** ** and for those with education level not age appropriate**	No gap	1.0	1.0
	Years of gap	0.996 (0.985 – 1.007)	0.992 (0.978 – 1.003)
**Participates in labor force**	No	1.0	1.0
	Yes	0.919 (0.598 – 1.161)	1.120 (0.813 – 1.372)
**Attends school and participates in labor force**	No	1.0	1.0
	Yes	1.005 (0.895 – 1.018)	0.968 (0.602 – 0.999)***
**Lives with a partner**	No	1.0	1.0
	Yes	0.760 (0.362 – 1.189)	0.989 (0.597 – 1.394)
**Has close friend they can count on**	No	1.0	1.0
	Yes	0.721 (0.328 – 1.337)	0.990 (0.458 – 1.854)
**Drinks alcohol**	No	1.0	1.0
	Yes	1.099 (0.848 – 1.240)	1.042 (0.693 – 1.241)
**Smokes**	No	1.0	1.0
	Yes	1.174 (1.034 – 1.226)**	1.173 (0.979 – 1.251)*
**Reported STI symptoms**	No	1.0	1.0
	Yes	0.969 (0.656 – 1.004)	1.023 (0.968 – 1.030)
**Used condom in last sex**	No	1.0	1.0
	Yes	1.151 (0.916 – 1.269)	1.113 (0.870 – 1.232)
**Reported compensated sex**	No	1.0	1.0
	Yes	1.014 (1.006 – 1.015)**	1.049 (0.012 – 1.052)**
**Geographical area of residence**	Center	1.0	1.0
	Gulf	1.274 (0.984 – 1.436)*	1.710 (1.291 – 1.906)***
	North	1.217 (0.744 – 1.391)	0.911 (0.462 – 0.999)*
	Pacific, North	0.944 (0.505 – 1.035)	1.040 (0.968 – 1.058)
	Pacific, Central	0.954 (0.723 – 1.037)	1.093 (0.995 – 1.128)*
	Pacific, South	1.019 (0.817 – 1.080)	1.151 (1.023 – 1.190)**
	Yucatan peninsula	0.976 (0.792 – 0.998)***	1.005 (0.915 – 1.014)
N^c^		1177	713

^a ^Estimated using the formulae PR = OR/ [(1-P0)+(P0*OR)] as suggested by Zhang & Yu [18]. ^b ^Confidence intervals were estimated reflecting design effect. ^c ^Number of observations is less than in descriptive because of missing values. ***P-value < 1%; **P-value < 5%; * P-value < 10%
