# Supplementary material for: Risk behaviors of 15–21 year olds in Mexico lead to a high prevalence of sexually transmitted infections: results of a survey in disadvantaged urban areas
Source: BMC Public Health. 2006 Feb 27;6:49. doi: 10.1186/1471-2458-6-49 (PMC1409781; doi:10.1186/1471-2458-6-49)
Supplement: Additional File 1 — Survey questionnaire. The English translation of the questionnaire is presented in a PDF file. [file 1471-2458-6-49-S1.pdf]

Survey of Urban Households 2001S

Youth questionnaire (10 to 21 years)

Copy the number
of the sticker of the page |__||__|__|__||__|__|__||__|

Stick the household number here

**Registration number of the interviewee** |___||___|		
**Name:**		
**Date of Birth** |__|__| |__||__| |__||__| Day Month YEAR		
**Sex:** |___| Male .... 1<br>Female 2	**Age:** |___|___| Years	**civil status:** (transcribe code) |___||___|

VISITS OF THE INTERVIEWER	1	2	3	4
DATE	|__|__| |__||__| |__||__| DAY MONTH YEAR	|__|__| |__||__| |__||__| DAY MONTH YEAR	|__|__| |__||__| |__||__| DAY MONTH YEAR	|__|__| |__||__| |__||__| DAY MONTH YEAR
NAME OF THE INTERVIEWER				
NUMBER OF THE INTERVIEWER	|__||__||__||__|	|__||__||__||__|	|__||__||__||__|	|__||__||__||__|
TIME STARTED	|__||__||__||__|	|__||__||__||__|	|__||__||__||__|	|__||__||__||__|
TIME ENDED	|__||__||__||__|	|__||__||__||__|	|__||__||__||__|	|__||__||__||__|
DURATION	|__||__||__| min	|__||__||__| min	|__||__||__| min	|__||__||__| min
NUMBER OF THE PERSON ANSWERING	|___||___|	|___||___|	|___||___|	|___||___|
RESULTS	|__||__|	|__||__|	|__||__|	|__||__|

*Results codes

01 Interview complete
02 Interview incomplete
05 They refused (why?)
09 Other (Note on observations)

NOTE REASONS IN TEXT

OBSERVATIONS

	SUPERVISED BY	CRITICADO BY	CODED BY	TAPED BY
NAME				
NUMBER	|__||__||__|	|__||__||__|	|__||__||__|	|__||__||__|
DATE	|__|__| |__||__| |__||__| DAY MONTH YEAR	|__|__| |__||__| |__||__| DAY MONTH YEAR	|__|__| |__||__| |__||__| DAY MONTH YEAR	|__|__| |__||__| |__||__| DAY MONTH YEAR

Good morning (afternoon), my name is ________________ and I work for the National Institute of Public Health. We are visitng households to speak about health, education, nutrition and some other things related to these issues. Your responses will be a great help for this study. The information that you give us is confidential and will only be used for statistical research and none of the results presented will make reference to any people in particular. You do not have to respond if you your don't wish to and this won't affect you in any way. Also, at any moment you can decide that you do not want to continue anymore or you can also tell us if you don't want to respond to certain questions. The information that you give us will help improve social programs related to heatlh and we are going to give you information about your health status.. Do you agree to answer the questionnaire?

Interviewer signature that the interviewee agreed to answer the questionnaire.

I. EDUCATION

Question	Response	SKIP TO
1. What is the maximum level of school that you have finished?<br><br>**CIRCLE THE LAST YEAR COMPLETED**	Never gone to school ....66<br>None ....00<br>Pre-school or kindergarten ....01<br>Elementary ....02<br>Secondary ....03<br>Preparatory ....04<br>Normal Basic ....05<br>Technical or commercial degree ....06<br>Bachelor's Degree ....07<br>Postgraduate ....08<br>DON'T KNOW ....98<br>NO RESPONSE 99	➔ 25<br><br><br><br><br><br><br><br><br>➔ 25<br>➔ 25
2. What is the last grade in school you passed?<br><br>**NOTE THE NUMBER OF THE LAST GRADE (YEAR) PASSED**	Grade |___|___| DON'T KNOW ....98<br>NO RESPONSE .... 99<br><br>**ASK THE NEXT QUESTION ONLY IF CODE06 OF QUESTION 1 WAS MARKED, IF NOT, SKIP TO QUESTION 4**	
3. How many prerequisites were required to get into this degree program?<br><br>**ONLY FOR THOSE THAT ANSWERED TECHNICAL OR COMMERCIAL DEGREE IN QUESTION 1**	None .... 1<br>Primary Completed ....2<br>Secondary Completed ....3<br>Preparatory Completed ....4<br>DON'T KNOW ....8<br>NO RESPONSE 9	
4. Are you currently attending school?	Yes .... 1<br>No ....2<br>DON'T KNOW ....8<br>NO RESPONSE 9	➔ 6<br><br>➔ 25

5. At what age did you leave school?	Age |___||___| DON'T KNOW .... 98<br>NO RESPONSE 99	→ 25
6. What is the name and location of the school that you attend?	Name<br><br>Locality/colony<br>Municipality<br><br>Zip code	
7. The school that you attend is ...	Public ?........ 1<br>Private non - religious?........ 2<br>Private religious?........ 3<br>DON'T KNOW .... 8<br>NO RESPONSE 9	
8. In what turn do you attend school?	Morning .... 1<br>Afternoon .... 2<br>Night .... 3<br>DON'T KNOW .... 8<br>NO RESPONSE 9	
9. Do you use transportation to get to school?	Yes .... 1<br>No .... 2<br>DON'T KNOW .... 8<br>NO RESPONSE 9	
10. How much time do you take to get to school?	hours |___| minutes|___||___| <br>DON'T KNOW .... 8 .... 98<br>NO RESPONSE 9 99	
11. On an average day, how many hours per day do you spend at school?	Hours per day |___|___|.|___| DON'T KNOW .... 98.8<br>NO RESPONSE 99.9	
12. In the past four weeks, how many days have you missed school?<br>**INTERVIEWER: THE RESPONSE CANNOT BE MORE THAN 24 DAYS**<br>**NO PUEDE SER MAYOR A 24 DÍAS**	Days |___|___| <br>Did not miss........ 00<br>DON'T KNOW .... 98<br>NO RESPONSE 99	
13. Outside of school how many hours a week do you spend studying and doing homework?	Hours |___||___| <br>DON'T KNOW .... 98<br>NO RESPONSE 99	

14. In your last report, what grade did you receive for math ?<br>**IF THE PERSON IS SI LA PERSONA IS IN PREPARATORY OR BACHELORS ASK ABOUT CÁLCULUS OR ÁLGEBRA, WHICH ARE EQUIVALENT TO MATH**	Math |___||___| Not applicable....00<br>DON'T KNOW ....98<br>NO RESPONSE 99	
15. ¿And what about your grade in Spanish?<br>**IF THE PERSON IS SI LA PERSONA IS IN PREPARATORY OR BACHELORS ASK ABOUT LITERATURE, WHICH IS EQUIVALENT TO SPANISH**	Spanish |___||___| Not applicable....00<br>DON'T KNOW ....98<br>NO RESPONSE 99	**SI LA PERSONA ESTA CURSANDO PREPARATORIA O BACHILLERATO PREGUNTAR SILLEVA LA MATERIA DE CÁLCULO O ÁLGEBRA LAS CUALES SON EQUIVALENTES A MATEMÁTICAS**
16. How many students are there in your classroom?	Students |___||___| DON'T KNOW ....98<br>NO RESPONSE 99	
17. How many teachers do you have?	Maestros |___||___| DON'T KNOW ....98<br>NO RESPONSE 99	
18. Do you have the necessary textbooks for this academic year?	Yes, all....1<br>Yes, some ....2<br>No ....3<br>DON'T KNOW ....8<br>NO RESPONSE 9	
19. Do your parents help you with your homework?	Yes ....1<br>No ....2<br>DON'T KNOW ....8<br>NO RESPONSE 9	
20. Have you ever stayed back in school?	Yes ....1<br>No....2<br>DON'T KNOW ....8<br>NO RESPONSE 9	**→ 22**

21. Which years did you repeat?<br><br>CIRCLE THE CODE(S) AND NOTE THE NUMBER OF TIMES	Times<br>**Primary**<br>First .... 01................|___| Second .... 02................|___| Third .... 03 ....|___| Fourth .... 04 ....|___| Fifth .... 05 ....|___| Sixth .... 06 ....|___| **Secondary**<br>First .... 07 ....|___| Second .... 08................|___| Third .... 09................|___| **Preparatory or bachillerate**<br>First .... 10................|___| Second .... 11................|___| Third .... 12................|___| <br>DON'T KNOW .... 98................|___| NO RESPONSE .... 99................|___|	
22. During the **last year** did you leave school for one month or more while you were inscirbed?	Yes ....1<br>No...2<br>DON'T KNOW ....8<br>NO RESPONSE 9	
23. Did you ever not attend in school and then return to school during another year?	Yes ....1<br>No...2<br>DON'T KNOW ....8<br>NO RESPONSE 9	➔ 25
24. How many years were you not signed up for school?	Years |___|___| <br>DON'T KNOW ....98<br>NO RESPONSE 99	

II. OCCUPATION The next questions refer to your daily activities.

25. During the **last week**, how many hours approximately did you dedicate to household tasks like taking care of children, elderly, or sick people without any payment?	Hours |___|___| Don't dedicate any time...00<br>DON'T KNOW ....98<br>NO RESPONSE 99	

26.	During the **last week**, how many hours approximately did you dedicate of offer free services to your community?	Hours |___|___| Don't dedicate any time....00<br>DON'T KNOW ....98<br>NO RESPONSE 99	
27.	During the **last week,** did you work in exchange for money to support your family or to cover some of your own expenses, at least for one hour?	Yes ....1<br>No....2<br>DON'T KNOW ....8<br>NO RESPONSE 9	➔ 32
28.	Even though you indicated to me that you didn't work in the last week, do you have some other job or employment through yourself? (Are you self-employed?)	Yes ....1<br>No....2<br>DON'T KNOW ....8<br>NO RESPONSE 9	➔ 32
29.	**Last week,** did you work without pay on family/friend's land for a family (or other) business?	Yes ....1<br>No....2<br>DON'T KNOW ....8<br>NO RESPONSE 9	➔ 31
30.	The person that you helped is...	A family member? ....1<br>not a family member? ....2<br>DON'T KNOW ....8<br>NO RESPONSE....9	➔ 32
31.	Can you tell me how you sustain yourself economically?<br>**CIRCLE ALL THAT APPLY**	Receive pension for WORK ....1<br>Receive money from renting a property or from business income ....2<br>Has savings ....3<br>Receive pension for widows, divorce, orphan ....4<br>Has scholarship....5<br>A family member or someone else sustains me....6<br>Other__________ 7<br>(specify)<br>DON'T KNOW ....8<br>**FOR ALL CASES SKIP TO 41**<br>NO RESPONSE....9	

		PRIMARY	SECONDARY	TERTIARY
32. In your WORK* (…) last week were you ...<br>**READ ALL OPTIONS AND NOTE THE RESPONSE IN THE CORRESPONDING COLUMN**<br>*** READ: PRINCIPAL WORK, SECONDARY WORK, TERTIARY WORK**	Boss?01<br>self-employed? 02<br>work for a commission, percentage ? 03<br>work for a fixed salary? 04<br>member of a cooperative? 05<br>work for a family business without pay? 06<br>work for a business (not family) without pay? 07<br>Other______________________________<br>(specify in the corresponding column)<br>DON'T KNOW 88<br>NO RESPONSE 99	|___|___| CODE<br>↓	|___|___| CODE<br>↓	|___|___| CODE<br>↓
33. What type of work does the business or the boss who you worked for in the past week do?	Agriculture, livestock, fishing, hunting 01<br>Informal Business 02<br>Construction 03<br>Education, Health or Social Services 04<br>Restaurant or hotel 05<br>Formal business (establishment) 06<br>Manufactures 07<br>Transport 08<br>Cultural, sporting , information services 09<br>Other services except government______________________<br>(specify in the corresponding column)<br>Government 11<br>DON'T KNOW 98<br>NO RESPONSE 99	|___|___| CODE<br>↓	|___|___| CODE<br>↓	|___|___| CODE<br>↓

<table>
<tr><td rowspan="4">34. Last week, how many hours did you work?

CIRCLE THE DAYS AND NOTE THE TOTAL NUMBER OF HOURS PER DAY</td><td>M</td><td>T</td><td>W</td><td>T</td><td>F</td><td>S</td><td>S</td><td>TOTAL 1</td><td>TOTAL 2</td><td>TOTAL 3</td></tr>
<tr><td>|__|__|</td><td>|__|__|</td><td>|__|__|</td><td>|__|__|</td><td>|__|__|</td><td>|__|__|</td><td>|__|__|</td><td>|__|__|</td><td></td><td></td></tr>
<tr><td>|__|__|</td><td>|__|__|</td><td>|__|__|</td><td>|__|__|</td><td>|__|__|</td><td>|__|__|</td><td>|__|__|</td><td></td><td>|__|__|</td><td></td></tr>
<tr><td>|__|__|</td><td>|__|__|</td><td>|__|__|</td><td>|__|__|</td><td>|__|__|</td><td>|__|__|</td><td>|__|__|</td><td></td><td></td><td>|__|__|</td></tr>
<tr><td rowspan="4">35. Normally, what days do you work and how many hours do you dedicate to your WORK?</td><td>M</td><td>T</td><td>W</td><td>T</td><td>F</td><td>S</td><td>S</td><td>T</td><td>W</td><td>T</td></tr>
<tr><td>|__|__|</td><td>|__|__|</td><td>|__|__|</td><td>|__|__|</td><td>|__|__|</td><td>|__|__|</td><td>|__|__|</td><td>|__|__|</td><td></td><td></td></tr>
<tr><td>|__|__|</td><td>|__|__|</td><td>|__|__|</td><td>|__|__|</td><td>|__|__|</td><td>|__|__|</td><td>|__|__|</td><td></td><td>|__|__|</td><td></td></tr>
<tr><td>|__|__|</td><td>|__|__|</td><td>|__|__|</td><td>|__|__|</td><td>|__|__|</td><td>|__|__|</td><td>|__|__|</td><td></td><td></td><td>|__|__|</td></tr>
</table>

	WORK		
	**PRIMARY**	**SECONDARY**	**TERTIARY**
36. In your **WORK**… from the last week, how much do they pay you and how often do you get paid?<br><br>**AMOUNT**<br><br>$98000 o more .... 98000<br>They pay by service, by piece.<br>or by finished WORK .... 99000<br>Did not work last week .... 00000<br>DON'T KNOW .... 98888<br>NO RESPONSE .... 99999<br><br>**PERIOD**<br><br>Every month .... 1<br>Every 15 days .... 2<br>Every week .... 3<br>Daily .... 4<br>Other period of pay__________ 5<br>(specify in the corresponding column)<br>DON'T KNOW .... 8<br>NO RESPONSE 9	$ |___|___|___|___|___| **AMOUNT**<br><br>|____| **CÓDE**<br><br>↓<br>**OTHER PERIOD**	$ |___|___|___|___|___| **AMOUNT**<br><br>|____| **CÓDE**<br><br>**OTHER PERIOD**<br>↓	$ |___|___|___|___|___| **AMOUNT**<br><br>|____| **CÓDE**<br><br>**OTHER PERIOD**<br>↓
37. Normally how much do you earn per month for your **WORK** ...?<br><br>**AMOUNT**<br><br>$98000 o más .... 98000<br>DON'T KNOW .... 98888<br>NO RESPONSE 99999	$ |___|___|___|___|___| ↓<br>**AMOUNT**	$ |___|___|___|___|___| ↓<br>**AMOUNT**	$ |___|___|___|___|___| ↓<br>**AMOUNT**

The next question applies only to those who answered "they get paid by service, work, piece or completed WORK" in question 36. For the rest, SKIP TO 39.

38. Normally how many **UNITS** (piece, service, work, etc.) Per week are made and sold and how much do you earn ?<br>**Specify WHAT UNITS ARE PRODUCED OR SOLD**			
	**UNITS**	**UNITS**	**UNITS**
	|___|___|___|___| **AMOUNT**	|___|___|___|___| **AMOUNT**	|___|___|___|___| **AMOUNT**
**UNITS**<br>9800 or more UNITS .... 9800<br>DON'T KNOW .... 9888<br>NO RESPONSE 9999	$ |___|___|___|___|___| **AMOUNT**	$ |___|___|___|___|___| **AMOUNT**	$ |___|___|___|___|___| **AMOUNT**
**AMOUNT**<br>$98000 or more .... 98000<br>DON'T KNOW .... 98888<br>NO RESPONSE 99999			

		PRINCIPAL	SECONDAR Y	TERTIARY
39. In your **WORK** (...) from **last week,** how many of the following do you have a right to?<br>**READ ALL OPTIONS AND CIRCLE THE ANSWERS OF THE INFORMANT**	Aguinaldo .... 01<br>Paid vacations .... 02<br>Utilities .... 03<br>IMSS .... 04<br>ISSSTE .... 05<br>SAR (Retirement savings system) .... 06<br>Living Credit .... 07<br>Private Health Insurance .... 08<br>Other________________ 09<br>(specify en la columna correspondiente)<br><br>DON'T KNOW .... 98<br>NO RESPONSE .... 99<br><br>Ninguno 00	01<br>02<br>03<br>04<br>05<br>06<br>07<br>08<br>09<br><br><br>98<br>99<br><br>↓<br>00	01<br>02<br>03<br>04<br>05<br>06<br>07<br>08<br>09<br><br><br>98<br>99<br><br>00<br>↓	01<br>02<br>03<br>04<br>05<br>06<br>07<br>08<br>09<br><br><br>98<br>99<br><br>00<br>**SKIP TO 41 41**
40. Besides the **WORK** (...) that we have already talked about Did you have any other work during the last **week?**	**NOTE THE CODE IN THE BOX**<br>Yes .... 1<br>No .... 2<br>DON'T KNOW .... 8<br>NO RESPONSE 9	|___| CODE<br><br>➔**Return to 32**	|___| CODE<br><br>➔**Return to 32**	

III. UTILIZATION OF HEALTH SERVICES

41. In the past four weeks, Did you go to ...		42. What type of insitution does (TYPE OFSERVICE) belong to?	43. In the last four weeks, how many times did you go to (TYPE OF SERVICE)?	44. How much was paid for the service recieved at (TYPE OF SERVICE)?	45. Did you or anyone in the household work in exchange for service or pay in kind for the attention received at (TYPE OF SERVICE)?
Yes ..... 1<br>No..... 2<br>DOESN'T KNOW ..... 8<br>NO RESPONSE ..... 9<br>**ASK FOR EACH SERVICE OR SKIP TO 51** →	**CODE**	Secretary of Health (SSA) ..... 1<br>IMSS ..... 2<br>IMSS Solidaridad ..... 3<br>ISSSTE ..... 4<br>Gobierno municipal ..... 5<br>Dispensario ..... 6<br>Otros ..... 7<br>DOESN'T KNOW ..... 8<br>NO RESPONSE ..... 9<br>→<br>**CODE**	DOESN'T KNOW ....98<br>NO RESPONSE ......99<br>→<br>**TIMES**	**INSIST TO OBTAIN AN ANSWER IN PESOS**<br>Nothing ....0000<br>They billed in piece ....0001<br>$ 9800 or more98<br>DON'T KNOW98<br>NO RESPONSE9999<br>→<br>**AMOUNT**	Yes..... 1<br>No ..... 2<br>DOESN'T KNOW 8<br>NO<br>→ **SKIP TO 48**<br>RESPONSE.. 9
a hospital for consultation (NOT TO STAY OVERNIGHT)?	1 2 8 9	|___|	|___|___|	$ |__|__|__|__|	1 2 8 9
a clinic or health center?	1 2 8 9	|___|	|___|___|	$ |__|__|__|__|	1 2 8 9
a private practice or specialist?	1 2 8 9		|___|___|	$ |__|__|__|__|	1 2 8 9
a midwife or health assistant?	1 2 8 9		|___|___|	$ |__|__|__|__|	1 2 8 9
an herbalist, traditional medicine doctor?	1 2 8 9		|___|___|	$ |__|__|__|__|	1 2 8 9
a pharmacy?	1 2 8 9		|___|___|	$ |__|__|__|__|	1 2 8 9

III. UTILIZATION OF HEALTH SERVICES				
46. ......How much time did the work last for payment of (TYPE DE SERVICIO)?<br><br>Did not work .... 00<br>DON'T KNOW .... 98<br>NO RESPONSE .... 99<br><br>→<br><br>**HOURS**	47. How much do you estimate the payment in kind to be for (TYPE OF SERVICE)?<br><br>Did not pay in kind..... 000<br>DON'T KNOW .... 988<br>NO RESPONSE .... 999<br><br>→<br><br>**AMOUNT**	48. The payment in cash, in kind, or in work that was made for (TYPE OF SERVICE), did it include prescribed medicine?<br><br>No .... 2<br><br>Yes....................... 1<br>Nothing was prescribed<br>DON'T KNOW...... 8<br><br>NO RESPONSE .. 9<br>**SKIP TO 50**<br>→	49. How much was paid for the prescribed medicine for (TYPE OF SERVICE)?<br><br>**INSIST TO OBTAIN A RESPONSE IN PESOS**<br>Nothing ....000<br>$ 980 or more980<br>DON'T KNOW988<br>NO RESPONSE99[illegible]<br><br>→<br><br>**AMOUNT**	50. How much was spent on the roundtrip trip to (TYPE OF SERVICE)?<br><br>**INSIST TO OBTAIN A RESPONSE IN PESOS**<br>Nothing............... . 000<br>$ 980 or more.......980<br>DON'T KNOW....988<br>NO RESPONSE...999<br>**SKIP TO THE NEXT SERVICE**<br>↓<br><br>**AMOUNT**
|____|____|	$ |___|___|___|	1 2 3 8 9	$ |___|___|___|	$ |___|___|___|
|____|____|	$ |___|___|___|	1 2 3 8 9	$ |___|___|___|	$ |___|___|___|
|____|____|	$ |___|___|___|	1 2 3 8 9	$ |___|___|___|	$ |___|___|___|
|____|____|	$ |___|___|___|	1 2 3 8 9	$ |___|___|___|	$ |___|___|___|
|____|____|	$ |___|___|___|	1 2 3 8 9	$ |___|___|___|	$ |___|___|___|
|____|____|	$ |___|___|___|	1 2 3 8 9	$ |___|___|___|	$ |___|___|___|

IV. UTILIZATION OF HOSPITALS

		SKIP TO
51. In the last year, did you stay at the hospital for one or more days?	Yes .... 1<br>No .... 2<br>DOESN'T KNOW .... 8<br>NO RESPONSE 9	→55
CIRCLE ALL THAT APPLY<br>52. The place where you were hospitalized, what type of institution did it belong to?	Secretary of Health (SSA) .... 01<br>IMSS .... 02<br>IMSS Solidaridad .... 03<br>ISSSTE .... 04<br>Municipal Government .... 05<br>Private Institution .... 06<br>Dispensary .... 07<br>Other type of institution?<br>_________ 08<br>(specify)<br>DON'T KNOW .... 98<br>NO RESPONSE 99	
53. In total, how many days were you hospitalized during the whole year?	**DAYS** |___|___|___| DOESN'T KNOW .... 988<br>NO RESPONSE 999	
54. What was the total expense for the hospitalization?	**AMOUNT $** |___|___|___|___|___| Did not spend anything .... 00000<br>$98000 or more .... 98000<br>DOESN'T KNOW .... 98888<br>NO RESPONSE 99999	

AFT ER THIS SECTION, THE FOLLOWING SECTION ONLY APPLIES TO THE PRIMARY INFORMANT

THANK ALL OTHERS AND FINISH UP WITH THEM

V. WELLNESS<br>Now I am going to ask you some questions about how you feel about various aspects in your life.		SKIP TO
55. Compared to the past, how happy do you feel with your present life?<br>**READ ALOUD ALL OPTIONS**	Not very happy .... 1<br>Happy enough .... 2<br>Very happy .... 3<br>NO RESPONSE 9	
56. In case of crisis (anything that could alter your life), how capable do you feel of dealing with it?<br>**READ ALOUD ALL OPTIONS**	Not capable at all .... 1<br>Up to a certain point .... 2<br>Very capable .... 3<br>NO RESPONSE 9	
57. How worried do you get about the relationship you have with your family?<br>**READ ALOUD ALL OPTIONS**	A little .... 1<br>Up to a certain point .... 2<br>A lot .... 3<br>NO RESPONSE 9	
58. Do you feel capable of responding to your obligations?	Yes, all my obligations .... 1<br>Some of my obligations .... 2<br>No, none of my obligations .... 3<br>NO RESPONSE 9	
59. Is there someone you can depend on for everything including personal and intimate issues?	Yes .... 1<br>No .... 2<br>DON'T KNOW .... 8<br>NO RESPONSE 9	

VI. VIOLENCE AND AGGRESSION		SKIP TO
60. Have you had any problems related to robbery, intimidation, aggression, or violence in the past 12?<br>**IF THE ANSWER IS NO OR NO RESPONSE: FOR WOMEN 12 AND OLDER SKIP TO 65. FOR ALL OTHERS SKIP TO 87**	Yes ..... 1<br>No ..... 2<br>NO RESPONSE 9	]<br>➔65
61. What happened?<br>**IF THERE IS MORE THAN ONE, REFER TO THE LAST ONE MENTIONED FOR THE FOLLOWING QUESTIONS**<br>**T LAST ONE** FOR T	Robbery, assault ..... 1<br>Aggression without any apparent motive ..... 2<br>Kidnapping ..... 3<br>Detention ..... 4<br>Discussion/altercation ..... 5<br>Sexual aggression ..... 6<br>Other _______ 7<br>(specify)<br>DON'T KNOW ..... 8<br>NO RESPONSE 9	
62. Where were you when it (MENTION THE VIOLENCE) happened?	Home ..... 1<br>School ..... 2<br>Work ..... 3<br>Public area ..... 4<br>Countryside ..... 5<br>Sporting facility ..... 6<br>Commercial establishment ..... 7<br>Other _______ 8<br>(specify)<br>NO RESPONSE 9	
63. Did you know the person/ people who hurt you?	Yes ..... 1<br>No ..... 2<br>I don't know ..... 3<br>NO RESPONSE 9	
**64. INTERVIEWER: Were there other people present during the interview of this section?**<br>**CIRCLE THE ANSWER AND IF THE ANSWER IS YES, WRITE THEIR NUMBER IN THE SPACE**	Yes ..... 1<br>Code |___|___| Code |___|___| Code |___|___| Code |___|___| No ..... 2<br>Another person that is not a part of this household 6	

THE FOLLOWING QUESTIONS ARE ONLY FOR WOMEN 12 YEARS AND OLDER.
FOR ALL OTHER, PLEASE SKIP TO 87

VII. REPRODUCTIVE HEALTH	SKIP TO

VII. REPRODUCTIVE HEALTH		SKIP TO
65. Have you ever been pregnant?	Yes .... 1<br>No .... 2<br>NO RESPONSE 9	] → 87
66. How many times have you been pregnant?	Number of times|___|___| DOESN'T KNOW .... 98<br>NO RESPONSE 99	
67. Have any of your children died?<br>**CIRCLE THE ANSWER AND IF YES, ASK: HOW MANY OF YOUR CHILDREN HAVE DIED?**	Yes .... 1<br>Number of children that have died |___|___| No .... 2<br>NO RESPONSE 9	
68. Are you currently pregnant?	Yes .... 1<br>No .... 2<br>DOESN'T KNOW .... 3<br>NO RESPONSE 4	→ 87
69. Have you been pregnant in the past 12 months?	Yes .... 1<br>No .... 2<br>DOESN'T KNOW .... 8<br>NO RESPONSE 9	→ 87
70. During this pregnancy, who examined you?<br>**CIRCLE ALL THAT APPLY**	Doctor .... 1<br>Nurse .... 2<br>Midwife .... 3<br>Community worker .... 4<br>Health promoter .... 5<br>Family or friend .... 6<br>No one .... 7<br>Other ______ 8<br>(Specify)<br>NO RESPONSE 9	] → 75<br>→ 75

71. Where did you get your first check up for this pregnancy?	**IMSS**<br>– Clinic or Hospital .... 01<br>– Clinic Solidaridad .... 02<br>**SSA**<br>– Health Center or Hospital .... 03<br>– Auxiliar de Salud .... 04<br>**ISSSTE** .... 05<br>**DIF** .... 06<br>Other government institutions .... 07<br>Clinic or Private Hospital .... 08<br>Home of midwife .... 09<br>Woman's home .... 10<br>Other<br>______ 11<br>(specify)<br>NO RESPONSE 99	
72. In what month of your pregnancy did you have a checkup for the first time?	Month |___|___| DOESN'T KNOW.................................. 98<br>NO RESPONSE 99	
73. How many times in total did you have check ups during this pregnancy?	NUMBER OF TIMES |____|____| DOESN'T KNOW.. 98<br>NO RESPONSE.. 99	
74. During the revisions...<br><br>**READ EACH OPTION AND CIRCLE**	**Yes No DN NR**<br><br>Did they give you a vaccine for tetanus? 1 2 8 9<br>Did they prescribe :<br>iron pills ? 1 2 8 9<br>vitamins? 1 2 8 9<br>Did they take your pressure? 1 2 8 9<br>Did they weigh you? 1 2 8 9<br>Did they recommend:<br>breastfeeding? 1 2 8 9<br>Did they speak with you about:<br>family planning? 1 2 8 9<br>Did they offer any contraceptives for after you give birth? 1 2 8 9	

Question	Response	Skip
75. Was the result of this birth (twins, triples, etc.)?	Yes .... 1<br>How many babies were born? |___| No .... 2<br>NO RESPONSE 9	
76. What was the result of your last pregnancy?<br>**FOR MULTIPLE BIRTHS CODE 1 IF ONE OR MORE CHILDREN LIVED, CODE 2 ONLY IF ALL CHILDREN DIED**	Born alive .... 1<br>Born dead .... 2<br>Miscarriage 3	Born dead, Miscarriage ➔ 87
77. When was the baby born (the babies)?	Day........ |___|___| Month .... |___|___| Year ....|___|___|___|___| DON'T KNOW........ 98<br>NO RESPONSE 99	
78. What is (are) the name of your son(s) and/or daughter(s)?<br>**NOTE THEIR CODE AND THEIR NAME. IF THE BABY DIED, CODE THEM AS 00**	Name<br>Code |___|___| Name<br>Code |___|___| Name<br>Code|___|___|	
79. Who attended to you during this birth?	Doctor .... 01<br>Nurse .... 02<br>Midwife .... 03<br>Community Auxiliary .... 04<br>Health Promoter .... 05<br>No one .... 06<br>Family .... 07<br>Other ____________________ 08<br>(specify)<br>DON'T KNOW........ 98<br>NO RESPONSE........ 99	

Question	Response	Skip
80. Where did you give birth?	**IMSS**<br>– Clinica or Hospital .... 01<br>– Clinic Solidaridad .... 02<br>**SSA**<br>– Health Center or Hospital .... 03<br>– Health Auxiliary .... 04<br>**ISSSTE** .... 05<br>**DIF** .... 06<br>Other government institutions .... 07<br>Clínic or Private Hospital .... 08<br>Midwife's home .... 09<br>Woman's home .... 10<br>Other ____________ 11<br>(specify)<br>NO RESPONSE 99	
81. How many days after birth was your son(s)/daughter(s) weighed?	Days |___|___| He/she was not weighed .... 1<br>DON'T KNOW .... 2<br>NO RESPONSE 9	➔ 83
82. How much did (NAME) weigh at birth?<br>**MAKE SURE TO KEEP THE SAME ORDER AS IN 78**	Weight in kilograms |___|.|___|___|___| Weight in kilograms |___|.|___|___|___| Weight in kilograms |___|.|___|___|___| DON'T KNOW .... 9888<br>NO RESPONSE 9999	
83. Have you ever breastfed your child even for a short period?	Yes .... 1<br>No .... 2<br>DON'T KNOW .... 8<br>NO RESPONSE 9	] ➔ 87
84. How many months did you breastfeed for?<br>**MAKE SURE TO KEEP THE SAME ORDER AS IN 78**	Months |___|___| Months |___|___| Months |___|___| Died while breastfeeding .... 77<br>Still breastfeeding .... 78<br>DON'T KNOW .... 98<br>NO RESPONSE 99	

85. How old was your child when you began to give him/her other types of food (including formula or cow's milk)?<br>**MAKE SURE TO KEEP THE SAME ORDER AS IN 78**	Months |___|___| Months |___|___| Months|___|___| Less then a month .... 00<br>Died after alimentation .... 04<br>Only breast milk .... 05<br>DON'T KNOW .... 98<br>NO RESPONSE 99	
86. Before beginning to give him/her other food or milk on a daily basis, did you first give your child other liquids?<br>**CIRCLE ALL MENTIONED**	Yes<br>Tea .... 1<br>Water .... 2<br>Sugar water .... 3<br>Otro<br>______________ 4<br>(specify)<br>No .... 5<br>Don't remember 9	

VIII. ADDICTIONS (FOR ALL TEENAGERS)

		SKIP TO
87. Have you ever smoked a cigarette in your life even if it was only one or two or even just a drag of a cigarette?	Yes .... 1<br>No .... 2<br>NO RESPONSE 9	] (No, NO RESPONSE) → 93
88. Do you currently smoke?	Yes .... 1<br>No .... 2<br>NO RESPONSE 9	] (No, NO RESPONSE) → 93
89. How old were you when you began to smoke cigarettes regularly (at least one cigarette per month)?	NO RESPONSE .... 99<br>**YEARS**|___|___|	
90. During the week did you smoke.....	1 or 2 days? .... 1<br>3 or 4 days? .... 2<br>5 or 6 days ? .... 3<br>Daily? .... 4<br>Never .... 0<br>NO RESPONSE 9	
91. On a normal day, how many cigarettes do you usually smoke?	**NUMBER** |___|___| NO RESPONSE 99	
92. During the **last week** , how much money did you spend on cigarettes?	**AMOUNT** $ |___|___|___| More than $980 .... 980<br>NO RESPONSE 999	
93. Do you drink alcoholic drinks even if it is very occasionally?	Yes .... 1<br>No .... 2<br>NO RESPONSE 9	] (No, NO RESPONSE) → 101

VIII. ADDICTIONS (FOR ALL TEENAGERS)		SKIP TO
94. On average, how many beers, wine coolers, etc. do you drink during the week?	**BEERS** |____|____| Does not drink .... 00<br>NO RESPONSE 99	
95. On average, how many cups of tequila , rum, aguardiente, etc. do you drink during the week?	**CUPS** |____|____| Does not drink .... 00<br>NO RESPONSE 99	
96. In the last month, how often did you get drunk?	Did not drink last month .... 1<br>One time .... 2<br>2 or 3 times in the past month .... 3<br>One or more times per week .... 4<br>NO RESPONSE 9	
97. During the **last week** , how much money did you spend on alcoholic drinks?	**AMOUNT** $ |___|___|___| More than $980 .... 980<br>NO RESPONSE 999	
98. During the past four weeks, was there any occasion that you couldn't take care of your daily activities due to alcohol?	Yes .... 1<br>No .... 2<br>NO RESPONSE 9	
99. Does alcohol ever make you feel violent?	Yes .... 1<br>No .... 2<br>NO RESPONSE 9	] → 101
100. How frequently would you say this happens?	Rarely .... 1<br>Some time .... 2<br>Frequently .... 3<br>Almost always .... 4<br>NO RESPONSE 9	

IF AT LEAST ONE RESPONSE IS YES, CONTINUE. IF NOT THANK THEM AND END

101. Could you tell me if you have taken, used or tried......

	Yes	No	NR
**INHALANTS** (thinner, glue, paint)?	1	2	9
**MARIJUANA**?	1	2	9
**HALUCINOGENS** (mushrooms, peyote, mescaline, LSD)?	1	2	9
**COCAINE**	1	2	9
**AMPHETAMINES** (Stimulants for weight loss or to give energy)?	1	2	9
**SEDATIVES** (tranquilizers)?	1	2	9
**OPIATES** (heroine and morphine)?	1	2	9
**OTHER DRUGS**	1	2	9

(specify)

102. During the last four weeks, how many days have you consumed any of these substances?	Days |___|___| NO RESPONSE 99	➔ **END**
103. During the last week, how much money did you spend buying these substances?	AMOUNT $ |___|___|___| More than $980 .... 980<br>NO RESPONSE 999	
**THANK THEM AND END**		

**TO BE FILLED OUT BY THE INTERVIEWER**		
104. During the interview, how well did the informant understand the questions?	Very well .... 1<br>Well .... 2<br>OK .... 3<br>Very little 4	
105. How reliable do you think the responses were?	Very reliable .... 1<br>Reliable .... 2<br>More or less reliable .... 3<br>Not reliable 4	
106. During the interview, the interviewee:	Showed a lot of interest .... 1<br>Showed little interest .... 2<br>Showed apathy .... 3<br>Was hostile 4	

**OBSERVATIONS**

NUMERO DE RENGLON	ALL INDIVIDUALS 15 TO 21 YEARS				
	SEXUAL EXPERIENCE AND KNOWLEDGE OF CONTRACEPTIVE METHODS				
	27	28	29	30	31
	Do you know or have heard about any method to avoid pregnancy or not having babies?<br><br>Yes .... 1<br><br>No..................... 2<br><br>NO<br>**PASE A 29**<br><br>DON´T ANSWER 9	Which methods have you heard or do you know?<br><br>**DO NOT PROMT; CHECK THE FIRST 3 MENTIONED**<br><br>Pills. ....01<br>IUD..02<br>Injections.....................................03<br>Norplant ....04<br>Ovules, diafagram........................05<br>Condom ....06<br>Femele surgery 07<br>Male surgery.................................08<br>Billings, abstinence, temperature .09<br>Coitus interrumtus........................10<br>Other..11<br>DON´T KNOW ....98<br>DON´T ANSWER..........................99	At what age did you have your firsy sexual intercourse?<br><br>HAVEN´T HAD SEXUAL INTER-COURSE .... 000<br><br>DON´T KNOW 98<br>DON´T ANSWER 99<br><br>**PASE A 35**<br><br>**EDAD**	Have you had sexual intercourses in the last 4 weeks?<br><br>Yes..........................1<br><br>No ....2<br><br>DON´T ANSWER 99	Last time you have a sexual intercourse, what do you or your partner did to avoid a o pregnancy or a disease?<br><br>**DO NOT PROMT**<br><br>Pills. 01<br>IUD 02<br>Injections 03<br>Norplant 04<br>Ovules, diafagram 05<br>Condom 06<br>Femele surgery 07<br>Male surgery 08<br>Billings, abstinence, temperature 09<br>Coitus interrumtus 10<br>Other 11<br>DON´T KNOW 98<br>DON´T ANSWER................99
01	1 2 9	|__|__| |__|__| |__|__|	|__|__|	1 2 9	|__|__|
02	1 2 9	|__|__| |__|__| |__|__|	|__|__|	1 2 9	|__|__|
03	1 2 9	|__|__| |__|__| |__|__|	|__|__|	1 2 9	|__|__|
04	1 2 9	|__|__| |__|__| |__|__|	|__|__|	1 2 9	|__|__|
05	1 2 9	|__|__| |__|__| |__|__|	|__|__|	1 2 9	|__|__|
06	1 2 9	|__|__| |__|__| |__|__|	|__|__|	1 2 9	|__|__|
07	1 2 9	|__|__| |__|__| |__|__|	|__|__|	1 2 9	|__|__|
08	1 2 9	|__|__| |__|__| |__|__|	|__|__|	1 2 9	|__|__|
09	1 2 9	|__|__| |__|__| |__|__|	|__|__|	1 2 9	|__|__|
10	1 2 9	|__|__| |__|__| |__|__|	|__|__|	1 2 9	|__|__|

NUMERO DE RENGLON	ALL INDIVIDUALS 15 TO 21 YEARS			
	SEXUAL EXPERIENCE AND KNOWLEDGE OF CONTRACEPTIVE METHODS			
	32	33	34	35
	Who decided to use (METHOD) at that time?<br><br>She ....1<br>He...2<br>Both ....3<br>DON´T KNOW 8<br>DON´T ANSWER 9<br>→	Some adolescentes have sexual intercourses to get money, gifts, food or other favors. Do you have had a sexual intercourse for some of these reasons?<br><br>Yes...........................1<br>No ....2<br>**PASE A 35**<br>→<br>DON´T ANSWER 9	When was the last time you had a sexual intercourse to get money, gisft or food?<br><br>Last week.......................................1<br>Wihtin last 4 weeks ....2<br>Wihtin last 6 months ....3<br>Wihtin last year ....4<br>More than a year ago......................5<br>DON´T ANSWER 9<br>→	In the last 12 months, have you had any of the following...<br><br>genital warts?..1<br>gonorrhea?..2<br>syphilis? ....3<br>Chlamydia? ....4<br>genital herpes? ....5<br>Other STI? ....6<br>None ....7<br>DON´T KNOW 8<br>DON´T ANSWER 9<br>→
01	1 2 3 8 9	1 2 9	1 2 3 4 5 9	1 2 3 4 5 6 7 8 9
02	1 2 3 8 9	1 2 9	1 2 3 4 5 9	1 2 3 4 5 6 7 8 9
03	1 2 3 8 9	1 2 9	1 2 3 4 5 9	1 2 3 4 5 6 7 8 9
04	1 2 3 8 9	1 2 9	1 2 3 4 5 9	1 2 3 4 5 6 7 8 9
05	1 2 3 8 9	1 2 9	1 2 3 4 5 9	1 2 3 4 5 6 7 8 9
06	1 2 3 8 9	1 2 9	1 2 3 4 5 9	1 2 3 4 5 6 7 8 9
07	1 2 3 8 9	1 2 9	1 2 3 4 5 9	1 2 3 4 5 6 7 8 9
08	1 2 3 8 9	1 2 9	1 2 3 4 5 9	1 2 3 4 5 6 7 8 9
09	1 2 3 8 9	1 2 9	1 2 3 4 5 9	1 2 3 4 5 6 7 8 9
10	1 2 3 8 9	1 2 9	1 2 3 4 5 9	1 2 3 4 5 6 7 8 9
